# Highly stable and reusable imprinted artificial antibody used for *in situ* detection and disinfection of pathogens†
†Electronic supplementary information (ESI) available: Experimental section and elaboration of the results. See DOI: 10.1039/c5sc00489f



**DOI:** 10.1039/c5sc00489f

**Published:** 2015-02-23

**Authors:** Zhijun Zhang, Yijia Guan, Meng Li, Andong Zhao, Jinsong Ren, Xiaogang Qu

**Affiliations:** a Laboratory of Chemical Biology and Division of Biological Inorganic Chemistry , State Key Laboratory of Rare Earth Resource Utilization , Changchun Institute of Applied Chemistry , University of Chinese Academy of Sciences , Changchun Institute of Applied Chemistry , Chinese Academy of Sciences , Changchun , Jilin 130022 , China . Email: xqu@ciac.ac.cn

## Abstract

Sandwich ELISA methods have been widely used for biomarker and pathogen detection because of their high specificity and sensitivity. However, the main drawbacks of this assay are the cost, the time-consuming procedure for the isolation of antibodies and their poor stability. To overcome these restrictions, we herein fabricated artificial antibodies based on imprinting technology and developed a sandwich ELISA for pathogen detection. Both the capture and detection antibodies were obtained *via* an *in situ* method, with simplicity, rapidity and low cost. The peroxidase mimics, the CeO_2_ nanoparticles, as signal generators were integrated with the detection antibody. The fabricated artificial antibodies exhibited not only natural antibody-like binding affinities and selectivities, but also superior stability and reusability. The detection limit was about 500 CFU mL^–1^, which is much lower than that of traditional ELISA methods (10^4^ to 10^5^ CFU mL^–1^). Furthermore, the capture antibody can disinfect pathogens *in situ*.

## Abstract 2

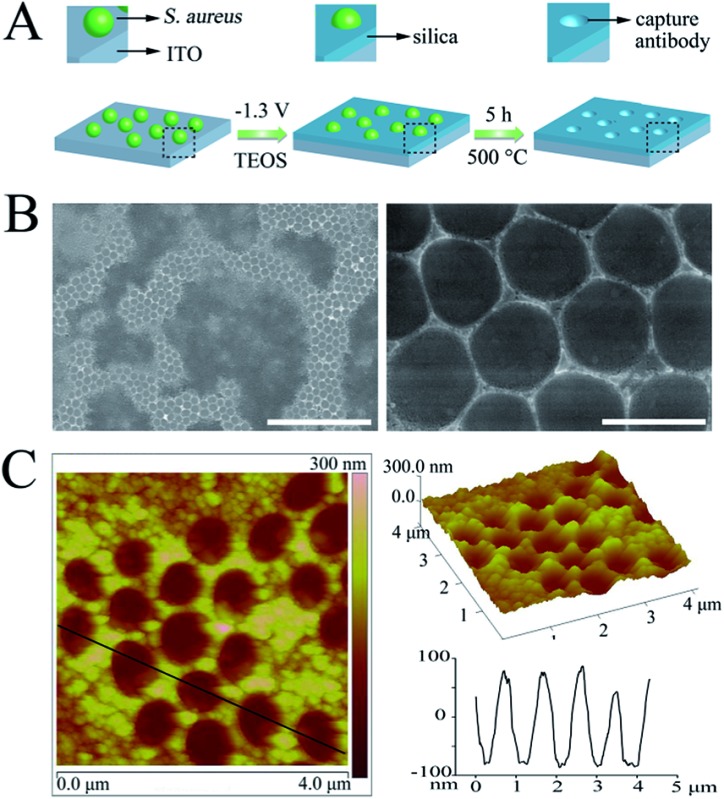
We fabricate artificial antibodies based on imprinting technology and develop a sandwich ELISA for pathogen detection.

## Introduction

The enzyme-linked immunosorbent assay (ELISA) is the current gold standard for clinical biomarker detection, as well as a robust approach for pathogen screening. Arguably, the most powerful ELISA format is the sandwich assay,^1–3^ in which the antigen is recognized by a couple of antibodies: a capture antibody (cAb) and detection antibody (dAb). The two antibodies recognition strategy endues the detection with high sensitivity and specificity.^4–6^ However, the main drawbacks of the sandwich ELISA are the cost, and the labor-intensive and time-consuming procedure for the screening of antibodies. In addition, owing to their easily disrupted stabilities, the natural antibodies usually cannot be reused. Furthermore, an appropriate enzyme should be linked to the dAb to clearly show the detection results. The complex process and expensive probes mean that the sandwich ELISA is not an optimal method for high-throughput screening of real samples. Therefore, the design and synthesis of artificial antibodies with easy availability and high stability, as alternatives to the natural antibodies, is urgently in demand for biodiagnostic applications.

Molecular imprinting technology (MIT) has been identified as a promising approach to synthesize artificial antibodies.^7–9^ The synthesized artificial antibodies exhibit a natural antibody-like binding affinity and selectivity. Intriguingly, they can even possess better characteristics than natural antibodies, including easy availability and operability, high stability to harsh chemical and physical conditions, and some even have superior reusability. To date, artificial antibodies against low molecular weight compounds^10–12^ and biological macromolecules^13–16^ based on MIT have been employed for a myriad of applications, such as separation,^17^ biomimetic catalysis,^18^ sensing,^19–22^ sewage treatment,^23–25^ enzyme inhibition^26–28^ and so on. In spite of promising prospects for molecular imprinting, it becomes more challenging as the target size increases, although nanoparticles^29^ and bioentities, such as viruses,^30–32^ microbes^33–38^ and mammalian cells,^39,40^ as templates for artificial antibody fabrication have recently been reported.

Herein, for the first time, we demonstrate a cell imprinted artificial antibodies-based sandwich ELISA for pathogen detection. Both the cAbs and dAbs were synthesized *via* an imprinting procedure. The cAbs were *in situ* fabricated on an indium tin oxide (ITO) conductive glass surface through an electrochemically assisted polycondensation method.^41,42^ The dAbs were synthesized using a sol–gel method with cerium dioxide nanoparticles (CeO_2_ NPs) integrated as artificial nanoenzymes. CeO_2_ NPs have recently been reported to possess excellent peroxidase-like activity toward the substrate 3,3′,5,5′-tetramethylbenzidine (TMB),^43–45^ which can be used to fabricate immunoassays.^46,47^ With the properties of easy availability, and superior stability and reusability, the fabricated artificial antibodies may circumvent the limitations of the natural antibodies and maintain natural antibody-like binding affinities and selectivities. What's more, with their conductivity properties, the cAbs can even disinfect the captured pathogen *in situ* by using an electrochemical technique.

## Results and discussion

### CAb fabrication

**Fig. 1:**
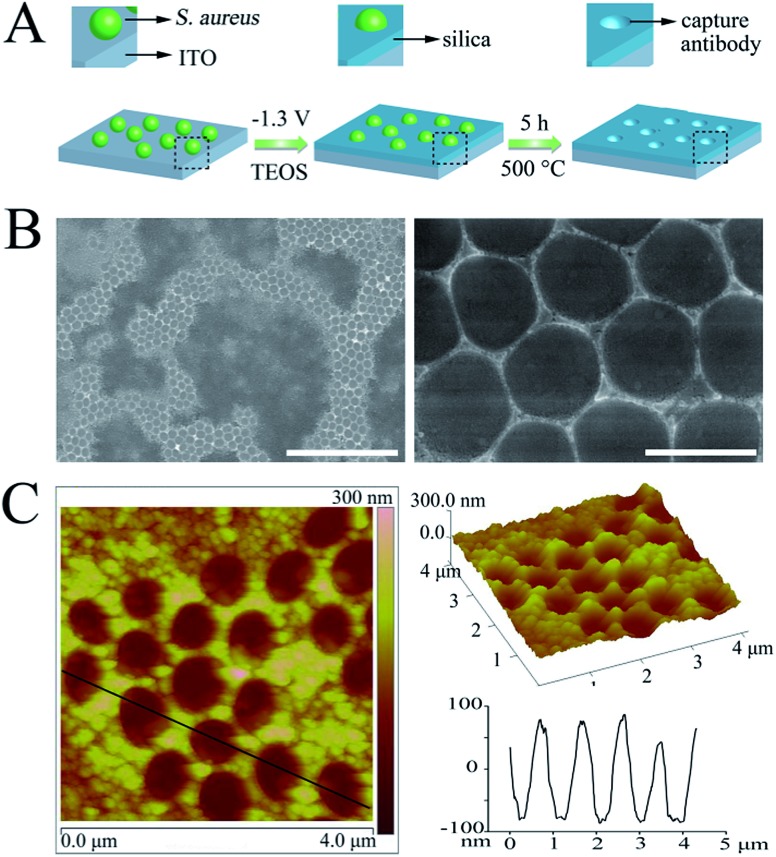
(A) Schematic diagram outlining the fabrication procedure for the cAbs, (B) SEM micrographs of the cAbs (scale bars = 10 μm and 1 μm, respectively), (C) 2D and 3D AFM images, and the corresponding height profiles of the cAbs.

As illustrated in Fig. 1A, the cAbs were fabricated on an ITO glass surface using *Staphylococcus aureus* (*S. aureus*) as a model of the target pathogen, which is one of the five most common causes of nosocomial infections. *S. aureus* was first immobilized on the aldehyde functionalized ITO glass surface through a Schiff base linkage (Fig. S1A and S1B†). Subsequently, a silica film was deposited on the electrode surface around the *S. aureus*, *via* an *in situ* electrochemically assisted polycondensation method (Fig. S1C†).^41,42^ Finally, the cAbs were obtained through a calcination treatment. After removal of the template, many regular cavity-cAbs were found to be scattered on the surface of the ITO glass (Fig. 1B and C). Images of the cavities at a higher magnification revealed their circular shape (Fig. 1B and C). The depths of the cavities were measured to be about 160 nm from the AFM image (Fig. 1C). Evidently, the three-dimensional spheroidal architecture of the template pathogen was imprinted well on the ITO glass surface. The fabrication procedure was also characterized using electrochemical methods (Fig. S2†).

### DAbs fabrication

**Fig. 2:**
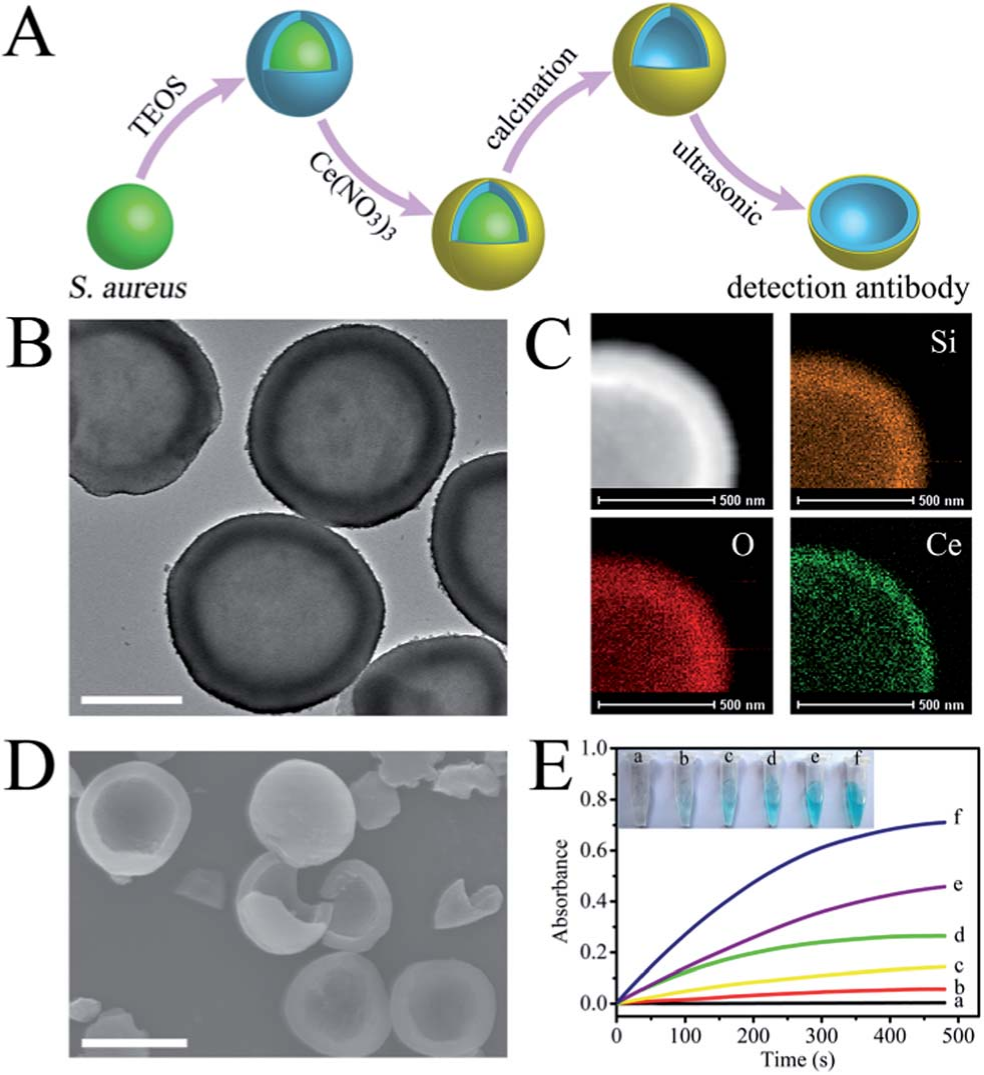
(A) Schematic representation of the synthesis of the dAbs; (B) TEM image of the hollow SiO_2_@CeO_2_ shells (scale bar = 500 nm); (C) dark-field TEM images and the corresponding TEM elemental mappings for the Si, O and Ce signals of the SiO_2_@CeO_2_ shell; (D) SEM image of the dAbs (scale bar = 1 μm); (E) time-dependent absorbance changes at 652 nm for TMB reaction solutions catalyzed by different concentrations of the dAbs ((a–f): 0, 2, 5, 10, 20, 50 μg mL^–1^).

The enzyme-linked dAbs were obtained through four simple steps: (i) *in situ* encapsulation of *S. aureus* with a silica shell, (ii) deposition of CeO_2_ NPs on the silica shell surface, (iii) calcination to remove the template and (iv) ultrasonic treatment to crush the hollow SiO_2_@CeO_2_ shells (Fig. 2A and S4†). Fig. 2B shows a typical TEM image of the hollow SiO_2_@CeO_2_ shells after removal of the template pathogen. The cavities of the hollow spheres were similar in size to *S. aureus*. Meanwhile, a thin CeO_2_ shell was found to be uniformly deposited on the hollow sphere surface (Fig. 2B and C). After a harsh ultrasonic treatment, the hollow spheres were cracked and cap-like dAbs were obtained (Fig. 2D). The empty cavities of the dAbs were found to maintain the size and shape of the original *S. aureus*. Altogether, these results confirmed that the shape and size of the template pathogen were preserved.

The enzyme that is linked to the dAb is critical for the assay because it will directly catalyze reaction of the substrate to produce a detectable signal. In the present work, CeO_2_ NPs were chosen as artificial nanoenzymes and integrated with the dAb. The oxidation of TMB by CeO_2_ NPs in the presence of H_2_O_2_ produced a blue color, with two absorbance bands at 370 and 652 nm.^43^ Fig. 2E exhibits the time-dependent absorbance change (at 652 nm) for different concentrations of the dAbs. The dAbs demonstrated a high catalytic activity toward the oxidation of TMB. Notably, even 2 μg mL^–1^ of the antibodies could produce a detectable signal within 10 min (Fig. 2E).

### Target pathogen recognition tests

**Fig. 3:**
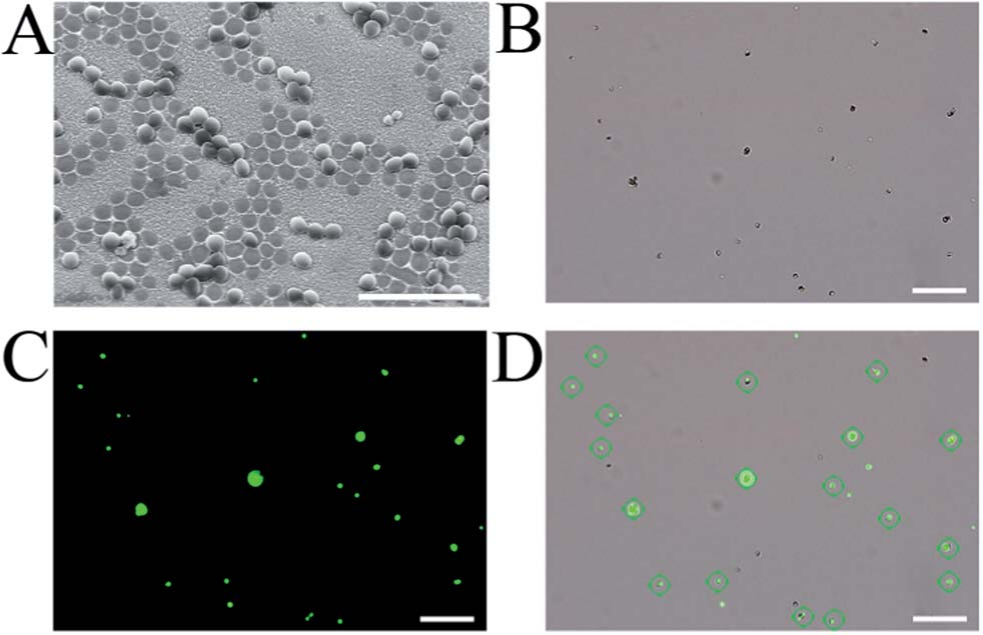
(A) SEM image of *S. aureus* captured by the cAbs (scale bar = 5 μm); (B) bright-field image of the mixture of dAbs and *S. aureus*, (C) fluorescence image revealing the positions of *S. aureus*, (D) an overlay of (B) and (C); the green circles mark the binding of *S. aureus* to the dAbs (scale bar = 10 μm).

Having successfully fabricated both the cAbs and dAbs, we next investigated the target pathogen recognition capacity of these antibodies. As shown in Fig. 3A, *S. aureus* was only found to be present on the imprinted cavities, and none was found in the non-imprinting area, indicating that *S. aureus* could be efficiently captured by the cAbs. Meanwhile, the non-target pathogens *Escherichia coli* (*E. coli*) and yeast cells were difficult to find on the plate (Fig. S5†). Even *Staphylococcus epidermidis* (*S. epidermidis*), which is similar in shape and size to *S. aureus*, was found to be much less captured by the cAbs (Fig. S7†). The recognition capacity of the dAbs was characterized using fluorescence microscopy. For better identification, *S. aureus* was stained with calcein-AM, to give a green fluorescence. Before photographing, the stained *S. aureus* and dAbs were dispersed in buffer and then moderately shaken for 10 min. As shown in Fig. 3B–D, most of the green fluorescence and crescent-shaped dAbs overlapped at the same spot, revealing that the dAbs were tightly bound to *S. aureus*. The binding specificity of the dAbs was also evaluated. Evidently, the dAbs could specifically bind to *S. aureus* over *E. coli*, yeast cells and *S. epidermidis* (Fig. S6 and S7†). Taken together, both the fabricated cAbs and dAbs could recognize target pathogens with high specificity. The target pathogen-like size and shape of the imprinted cavities may play an important role for the high recognition and selectivity capability.^35,36,40^ In addition, the imprinting of the surface chemistry of the pathogen at the molecular level would also contribute to the natural antibody-like properties.^33,34,40^

### Pathogen detection

**Fig. 4:**
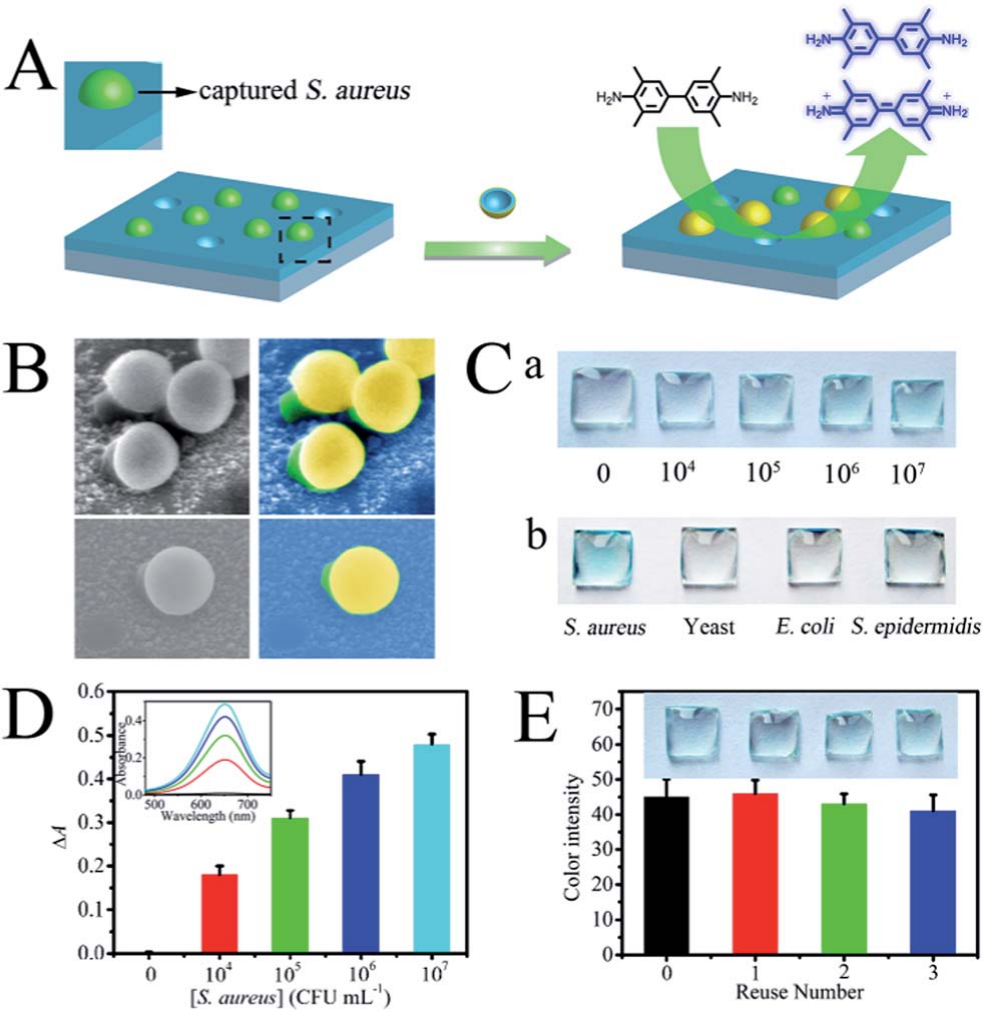
(A) Schematic illustration of the sandwich ELISA for *S. aureus* detection, (B) SEM images of the constructed sandwich ELISA (left) and the corresponding false color images (right), (C) images of the colorimetric detection of pathogens using the fabricated sandwich ELISA: (a) different concentrations of *S. aureus* and (b) different pathogens at 10^7^ CFU mL^–1^, (D) the corresponding absorbance values as determined from the absorption spectra of the test plates in Ca, (E) images of the reused antibodies-based sandwich ELISA for *S. aureus* (10^7^ CFU mL^–1^) detection (the colour intensity was measured for red in RGB format in Adobe Photoshop).

The target pathogen recognition capability of both the cAbs and dAbs, and the high catalytic activity of the dAbs, adequately meet the requirements for construction of a sandwich ELISA. Therefore, a sandwich ELISA based on these artificial antibodies was set up for *S. aureus* detection. A schematic representation of the sandwich ELISA format is presented in Fig. 4A. The target pathogens were first selectively captured by the cAbs-functionalized ITO glass, and subsequently the captured pathogens were recognized by the dAbs. Finally, the blue color was generated through the oxidation of TMB by the dAbs in the presence of H_2_O_2_. The constructed assay was characterized using SEM imaging. As shown in Fig. 4B, the sandwich structure was clearly observed, showing that *S. aureus* was captured by the cAbs and capped by the dAbs. The detection results are illustrated in Fig. 4C. No visible color was found on the control plate, while a distinguishable blue color appeared in the presence of *S. aureus* at 10^4^ CFU mL^–1^ (colony-forming units per milliliter). The color deepened gradually as the *S. aureus* concentration increased. UV-vis absorption spectra were used to quantify the results (Fig. 4D). The limit of detection was estimated to be about 500 CFU mL^–1^, which is much lower than that of traditional ELISA methods (10^4^ to 10^5^ CFU mL^–1^).^48^ The detection results could also be analyzed using image processing software (Adobe Photoshop)^49^ (Fig. S8†). The selectivity of the constructed sandwich ELISA was further investigated using non-target pathogens, *E. coli*, yeast cells and *S. epidermidis*, as controls. As presented in Fig. 4C, no obvious blue color appeared for the non-target pathogen detections, indicating the high specificity of the fabricated assay. The specific detection capability could be ascribed to the recognition specificity of the artificial antibodies. In addition, the two antibodies recognition strategy also plays an active role.

### Reusability tests

The reusability of natural antibodies is severely hampered by their poor stability. Herein, the artificial antibodies synthesized went through a high temperature calcination procedure, and they all exhibited high thermal stability. More importantly, both the cAbs and dAbs were found to be reusable through a simple calcination treatment. After calcination, the cAbs were found to maintain their original topography (Fig. S9†) and no obvious attenuation of the catalytic activity of the dAbs was observed (Fig. S10†). Even after treatment three times, the recovered sandwich ELISA could give a detection signal as high as 90% of the initial value (Fig. 4E), indicating their high reusability.

### Pathogen electrochemical disinfection

**Fig. 5:**
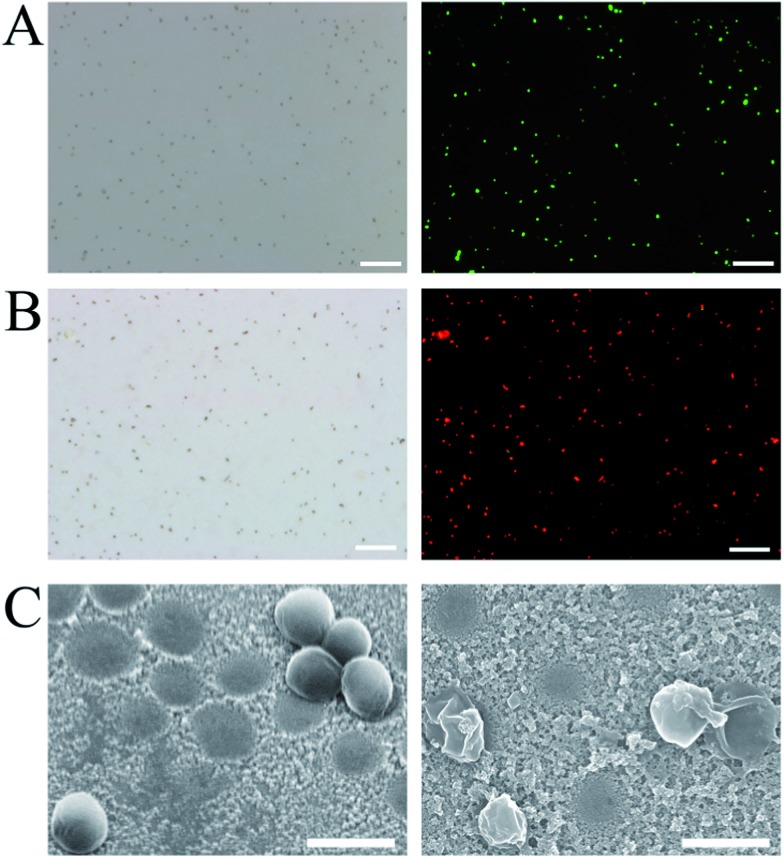
Optical images of the captured living *S. aureus* (A) and electrochemically disinfected *S. aureus* (B) stained by calcein-AM (green) and PI (red) (scale bar = 10 μm). (C) SEM images of the captured *S. aureus* cells before (left) and after (right) electrochemical treatment (scale bar = 1 μm).

For healthcare, it is more desirable that the detection and disinfection of pathogens can be realized at the same time. The fabricated cAbs could not only be used to construct a sandwich ELSA for pathogen detection, but could also be available to *in situ* electrochemically disinfect the captured pathogens. The fluorescent probes calcein-AM and propidium iodide (PI) were used to stain the living cells (green) and dead cells (red), respectively. As shown in Fig. 5A and B, after electrochemical treatment almost all of the *S. aureus* could be stained by PI, indicating that the *S. aureus* was disinfected. The electrochemical oxidation of intracellular coenzyme A (CoA), which is irreversibly converted to a CoA dimer by disulfide bond formation, and disruption of cell membranes have been generally considered to be responsible for disinfection activities.^50,51^ The cell membrane disruption effect was further demonstrated through SEM imaging. As seen in Fig. 5C, the morphology of the *S. aureus* was remarkably disrupted; many cells were found to be greatly shrunken and some had even collapsed.

Taken together, the fabricated artificial antibodies based on imprinting technology can be used for facile construction of a sandwich ELISA for the sensitive detection of pathogens. Compared with recently reported assays for the detection of pathogens (Table S1†), the most obvious advantage of the present method is that all the construction components are integrative, stable and reusable. In addition, apart from detection, the captured pathogens can even be *in situ* electrochemically disinfected.

## Conclusions

In summary, for the first time we have fabricated cell imprinted artificial antibodies to set up a sandwich ELISA for pathogen detection. Both the cAbs and dAbs were obtained *via in situ* methods, with simplicity, rapidity and low cost. The fabricated antibodies could be used without immobilization or an enzyme linkage procedure, which would streamline the process of sandwich ELISA construction. The constructed ELISA could be used for target pathogen detection with high sensitivity and selectivity. What's more, these artificial antibodies possess superior stability and reusability, which may circumvent the limitations of the natural antibodies. Besides, the cAbs can disinfect pathogens *in situ* by using an electrochemical technique. Thus, the present work may open a new avenue for designing stable and reusable artificial antibodies for immunoassays.
