# Supplementary material for: Highly stable and reusable imprinted artificial antibody used for in situ detection and disinfection of pathogens
Source: Chem Sci. 2015 Feb 23;6(5):2822–6. doi: 10.1039/c5sc00489f (PMC5489049; doi:10.1039/c5sc00489f)
Supplement: Supplementary file 1 [file SC-006-C5SC00489F-s001.pdf]

Electronic Supplementary Material (ESI) for Chemical Science.
This journal is © The Royal Society of Chemistry 2015

Supporting Information for

Highly stable and reusable imprinted artificial antibody used for in situ detection and disinfection of pathogens

*Zhijun Zhang, Yijia Guan, Meng Li, Andong Zhao, Jinsong Ren, and Xiaogang Qu**

Laboratory of Chemical Biology and Division of Biological Inorganic Chemistry, State Key Laboratory of Rare Earth Resource Utilization, Changchun Institute of Applied Chemistry, University of Chinese Academy of Sciences, Changchun Institute of Applied Chemistry, Chinese Academy of Sciences, Changchun, Jilin 130022, China.

Experimental Section

Materials: Ethanol, ammonium hydroxide (NH_3•H_2O), toluene and H_2O_2 were purchased from Beijing Chemicals (Beijing, China). All these reagents were of analytical reagent grade and were used as received without further purification. Cerium nitrate hexahydrate ($Ce(NO_3)_3$•$6H_2O$), hexamethylenetetramine (HMT), Tris(hydroxymethyl)aminomethane (Tris), (3-aminopropyl) triethoxysilane (APTES) and Tetraethoxysilane (TEOS) was purchased from Aladdin Chemistry Co., Ltd. (China). 3,3,5,5-Tetramethylbenzidine (TMB) was purchased from BBI (Ontario, Canada). Calcein-AM and propidium iodide (PI) were purchased from sigma. Nanopure water (18.2 MΩ; Millpore Co., USA) was used throughout the experiment.

General techniques: SEM images were obtained with a Hitachi-4800 FE-SEM. TEM images and high-angle annular dark-field scanning TEM (HAADF-STEM) were recorded using a FEI TECNAI G2 20 high-resolution transmission electron microscope operating at 200 kV. AFM measurements were performed using Nanoscope V multimode atomic force microscope (Veeco Instruments, USA). Cell imaging was performed with an Olympus BX-51 optical system microscope (Tokyo, 30 Japan) with a Blue filter. Pictures were taken with an Olympus digital camera. FITR analyze was carried out on a Bruker Vertex 70 FITR Spectrometer. UV-Vis spectroscopy spectra were recorded with a JASCO-V550 spectrometer.

Electrochemical measurements: Electrochemical measurements were all performed with a CHI 660B Electrochemistry Workstation (CHI, USA). A three-electrode setup was used with an ITO working electrode, a common Ag/AgCl reference and a Pt wire auxiliary electrodes. Cyclic Voltammetry (CV) and Electrochemical impedance spectroscopy (EIS) were performed in aqueous solution containing 5 mM $K_3[Fe(CN)_6]/K_4[Fe(CN)_6]$ (1:1) mixture with 0.1M KCl as the supporting electrolyte. The CV curves were recorded with a scan rate of 50 mv s^{-1} form -0.2 – 0.6 V. The impedance spectra were recorded within the frequency range of 10^{-1}-10^{5}Hz. The amplitude of the applied sine wave potential in each case was 5 mV.

Pathogen culture: Staphylococcus aureus (*S. aureus*), Staphylococcus epidermidis (*S. epidermidis*) and Escherichia coli (*E. coli*) were cultured in Luria-Bertani (LB) culture medium in the presence of ampicillin (50 μg mL^{-1}) and grown at 37 ^{o}C for 12 h under shaking. Yeast cells were cultured in a yeast-extract-peptone-dextrose (YPD) broth at 30 ^{o}C for 36 h under shaking. The mixture was washed with Tris buffer (10 mM, pH 7.4, 0.85% NaCl) twice and resuspended in the buffer. The optical density at 600 nm was measured with a GE Ultrospec 500 Pro Spectrophotometer.

Capture antibodies fabracation: ITO conductive glass were first NH_2 functionalized with APTES. Briefly, the cleaned ITO were immersed in a 5% APTES aqueous solution for an hour then dried and aged overnight in an oven at 80 ℃ before immersing in 5%

glutaraldehyde for 2h. Then *S. aureus* were immobilized on the aldehyde functionalized ITO surface through the schiff base linkage. After that, a thin silica film in situ deposited on the *S. aureus* modified ITO surface through electro-assisted deposition method.[1] A typical sol consisted of 20 mL of ethanol, 20 mL of an aqueous solution of 0.1 M $NaNO_3$ to which were added 200 mmol L^{-1} TEOS. HCl was added in order to reach a pH close to 3. A -1.3 V cathodic potential was applied for certain duration. The electrode was then quickly removed from the solution and immediately rinsed with distilled water. Finally the template pathogen were wholly removed and an transparent ITO plate was obtained by calcination in air condition at 500 ℃ for 5 h. Notably, the calcination treatment didn't change the surface topography of the ITO glass, but only exhibited a little influence on the electrical conductivity which would not affect the functions of the fabricated antibodies.

Detection antibodies fabracation: The *S. aureus* encapsulated with silica shell (*S. aureus*@SiO_2) were performed according to the previous report with slight modification.[2] Briefly, The cultured *S. aureus* were separated from the culture media by centrifugation at 3000 rpm for 10 min. After then the bacteria pellet was dispersed into pure water with a final optical density of 0.2 at 600 nm. To which APTES (with a final concentration of 1 mM) and TEOS (with a final concentration of 50 mM) were added, then agitated overnight at room temperature. The white precipitates were isolated and purified by centrifugation and washed with pure water then dried overnight at 50 ℃. Cerium dioxide nanoparticles (CeO_2 NPs) were

in situ deposited on the silica shell surface according to previous report with slight modification.[3] The obtained 10 mg *S. aureus*@SiO_2 microspheres and 25 mg $Ce(NO_3)_3$•$6H_2O$ were dispersed in 15 mL ethanol solution through an ultrasonic treatment. Subsequently, 10 mL deionized water containing 0.1 g HMT was added to the *S. aureus*@SiO_2 solution with ultrasonic vibrations for 15min. Then the mixture was stirred for 2 h in an oil bath maintained at 70 ℃. The products were isolated by centrifugation and dried overnight at 60 ℃. The template pathogens were removed by calcination in air condition at 500 ℃ for 6 h. Through calcintion the template could be completely removed with no organics left (Fig. S3†). Finally the cap-like detection antibodies were obtained by ultrasonic treatment with an ultrasonic homogenizer CP501 (Cole Parmer) for about 10 min (small fragments were also generated by ultrasonic treatment).

Target pathogen recognition test: The capture antibodies recognition capacity toward *S. aureus* was investigated by SEM imaging. 30 μL of *S. aureus* suspension (10^7 CFU mL^{-1}) was dropped on the ITO plate and kept under humidity for 2 h at room temperature and the *S. aureus* suspension was disturbed once every 30 min. After then, the plate was washed with Nanopure water and immersed in formaldehyde/glutaraldehyde solution for 30 min. Before SEM imaging the cells was dried slowly at room temperature. The same procedures were used to investigate the non-target pathogens *E. Coli*, yeast cells and *S. epidermidis* binding capability of the capture antibodies. The detection antibodies recognition capacity toward *S.*

aureus was investigated by imaging with fluorescence microscopy. In order to aid the visualization *S. aureus* were stained with calcein-AM (green fluorescence). Before photographing, the stained *S. aureus* and capture antibodies were dispersed in buffer and then moderately shaken for 10 min. The selectivity of the detection antibodies were evaluated by mixture of detection antibodies, *S. aureus* (calcein-AM stained), non-target pathogens *E. coli* and yeast cells or *S. epidermidis* in the same tube. In view of the similar shape and size of *S. aureus* and *S. epidermidis*, *S. epidermidis* were fixed with 70% ethanol and stained with PI to give a red fluorescence.

Detection antibodies catalysis activity test: The catalysis activity of the detection antibodies were carried out in time course mode by monitoring the absorbance change at 652 nm on a Jasco V550 UV-Vis spectrophotometer.[4] Experiments were carried out using 5 mM H_2O_2 in a reaction volume of 500 mL buffer solution (pH 4.0, 25°C) with 800 μM TMB, and in the presence of a series of concentrations of detection antibodies.

Pathogen detection: The capture antibodies functionalized ITO glass was first cut into a certain size (7×7 mm^2). 30 μL of different concentrations of pathogens was dropped on to the plate and kept under humidity for 2 h at room temperature (disturbed once every 30 min). After being washed with nanopure water, 30 μL of detection antibodies (2 mg mL^{-1}) was dropped on to the plate surface and kept under humidity for anthor 2 h at room temperature

(disturbed once every 30 min). Finally, after being washed with nanopure water, 30 μL of the chromogenic buffer (pH 4.0) containing 5 mM H_2O_2 and 800 μM TMB were dropped, and kept under humidity for 30 min. The images were then captured using a digital camera and the average color intensity at the center (3×3 mm^2) was determined using Adobe Photoshop CS using the Red channel of the RGB-mode. For the UV–vis absorption spectra measurement, a larger capture antibodies functionalized ITO plate (12×12 mm^2) was used for construction the ELISA to collect enough TMB solution.

Reusability tests: For the capture antibodies, the used ITO plate was calcined in air condition at 500 ℃ for 5 h then an ultrosonic treatment to separate out the detection antibodies. For the detection antibodies, in view of the low dose used each time, we used a succenturiate treatment to test the rusabiity. Briefly, fresh fabricated detection antibodies (2 mg mL^{-1}) were mixed with *S. aureus* (10^7 CFU mL^{-1}) and shook for 10 min. Then a chromogenic buffer was added with a final concentration of 5 mM H_2O_2 and 800 μM TMB. After keeping for 30 min, the detection antibodies and *S. aureus* were separated and calcined in air condition at 500 ℃ for 5 h. Both the capture antibodies and detection antibodies were retreated for three times. And the each time treated antibodies were usd to set up sandwich ELISA for *S. aureus* detection (10^7 CFU mL^{-1}).

Pathogen electrochemical disinfection: The electrochemical inactivation experiments were performed in a 10 mM PBS pH 7.4 electrolyte containing 155 mM NaCl with the pathogens captured ITO plate as the anode. An anodic potential at 1.8 V vs. Ag/AgCl was applied for 10 min. After that the plate was washed with the buffer, and stained with Calcein-AM and PI for optical imaging. For SEM imaging the plate was immersed in formaldehyde/glutaraldehyde solution for 30 min and dried slowly at room temperature after washing with Nanopure water.

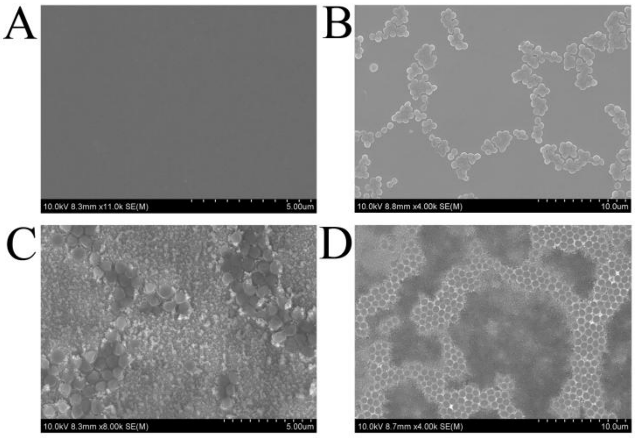

Fig. S1 SEM micrographs of the construction of capture antibodies: A) aldehyde functionalized ITO plate, B) *S. aureus* immobilized ITO plate, C) silica coated ITO plate and D) the obtained capture antibodies after calcination treatment.

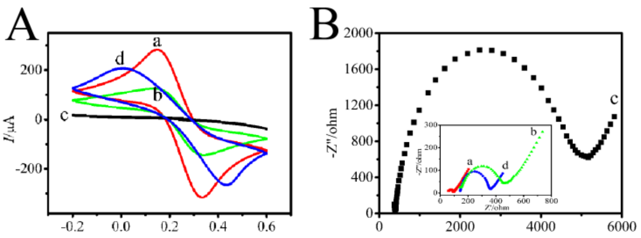

Fig. S2 Cyclic voltammograms (A) and Nyquist plots (B) of the ITO plate: a) aldehyde functionalized ITO plate, b) *S. aureus* immobilized ITO plate, c) silica coated ITO plate and d) the obtained capture antibodies after calcination.

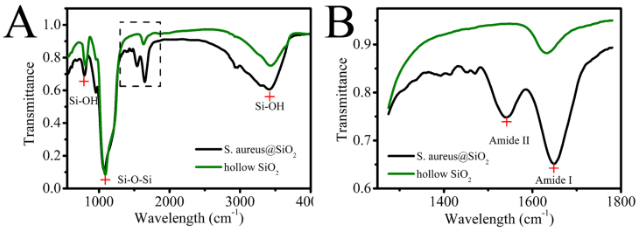

Fig. S3 FT-IR spectra of *S. aureus*@SiO_2 (black) and *S. aureus*@SiO_2 after calcination (green).

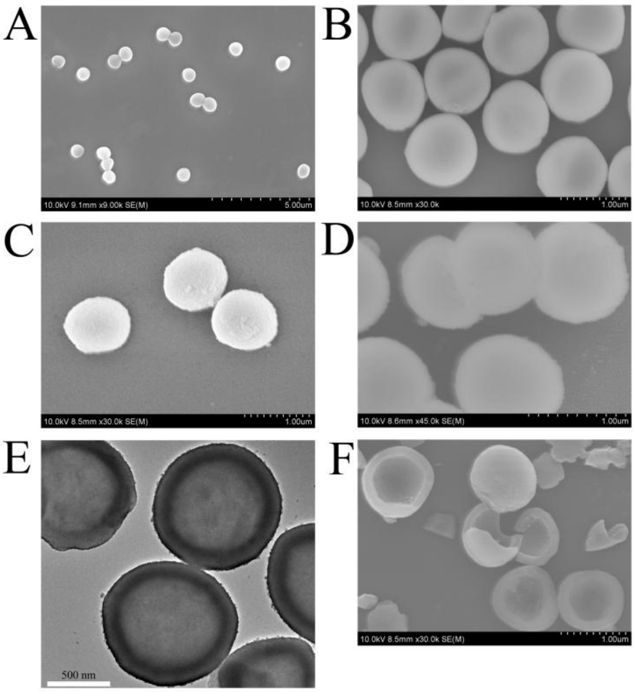

Fig. S4 Images of the fabrication of the detection antibodies: A) SEM image of *S. aureus*, B) SEM image of *S. aureus*@SiO_2, C) SEM image of *S. aureus*@SiO_2@CeO_2, D) SEM image of hollow SiO_2@CeO_2, E) TEM image of hollow SiO_2@CeO_2 and F) SEM image of capture antibodies.

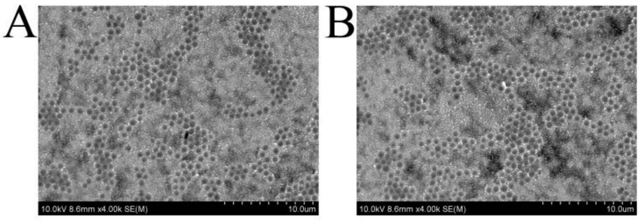

Fig. S5 SEM images of *E. coli* (A) and Yeast cells (B) captured by the capture antibodies functionalized ITO plate.

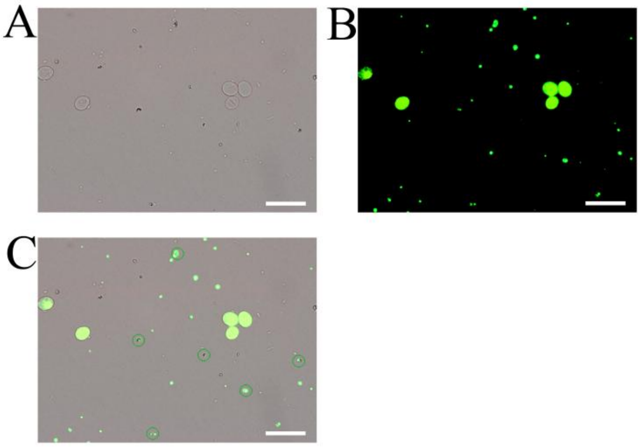

Fig. S6 The selective binging of *S. aureus* by the detection antibodies: A) bright-field image of the mixture of detection antibodies, *S. aureus*, *E. Coli* and Yeast cells, B) fluorescent image revealing the positions of *S. aureus*, C) overlay of B and C (The binding of detection antibody to *S. aureus* is indicated by green circle). Scale bar = 10 µm.

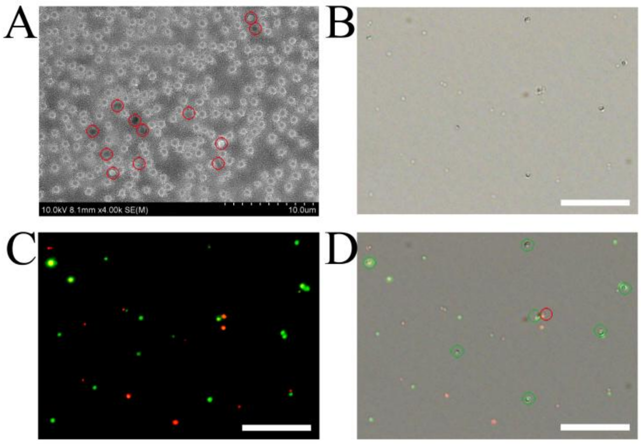

Fig. S7 SEM images of *S. epidermidis* (A) captured by the capture antibodies functionalized ITO plate (red circles mark the *S. epidermidis* positions). The selective binging of *S. aureus* by the detection antibodies in the presence of *S. epidermidis*: B) bright-field image of the mixture of detection antibodies, *S. aureus* and *S. epidermidis*, c) fluorescent image revealing the positions of *S. aureus* (green fluorescence) and *S. epidermidis* (red fluorescence), D) overlay of C and D (The binding of detection antibody to *S. aureus* is indicated by green circle and *S. epidermidis* by red circle). Scale bar = 10 μm.

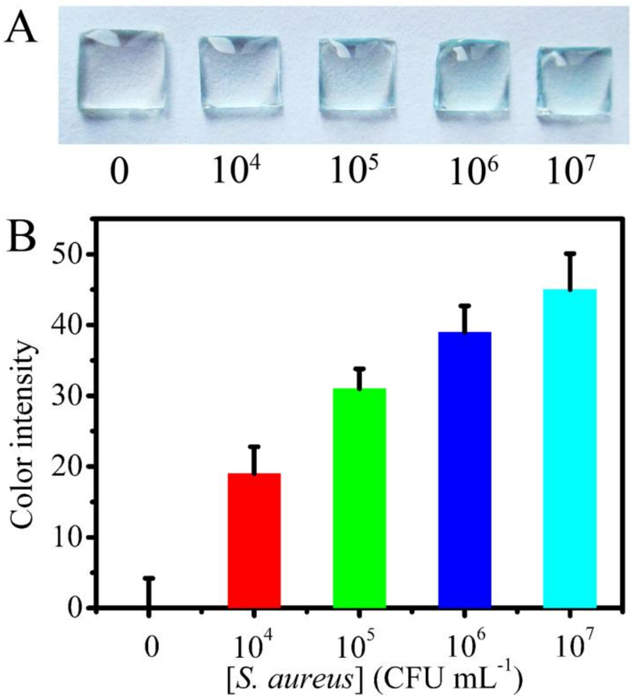

Fig. S8 A) Images of the colorimetric detection of *S. aureus* by the fabricated sandwich ELISA, B) graph of the corresponding average intensity of color measured in Red in RGB format in Adobe Photoshop from the test plates in A.

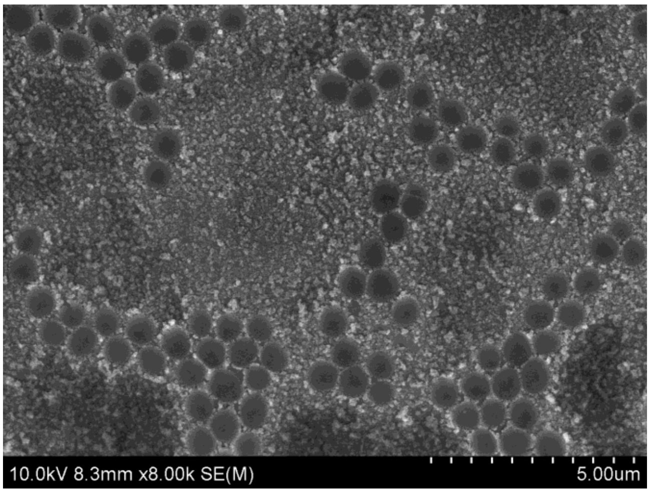

Fig. S9 SEM micrograph of the capture antibodies after calcination for three times.

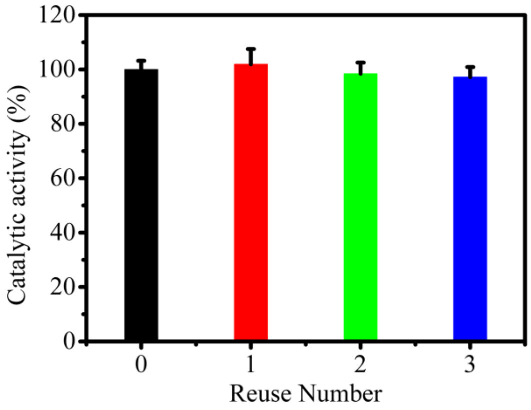

Fig. S10 TMB catalytic oxidation activities of the dAbs after recalcination.

Table S1 Comparison of the recently reported representative assays for the detection of bacteria

Method	Component	Bacterial strain	Detection range (CFU mL^{-1})	Limit of detection (CFU mL^{-1})	Visible to the naked eye	Signal on	Reusa-ble	Bacter-icidal effect	Ref
Colorimetric	AuNPs, β-galactosidase	*E. coli*	10^2-10^7	10^2	+	+	-	-	**5**
Fluorescence imaging	Dendronized polymers	*E. coli*	10^5-10^7 bacteria		-	+	-	-	6
Luminescent	HSA-AuNCs	*S. aureus*, MRSA	10^6-10^9	10^6	+	+	-	-	7
Dark-field imaging	AuNPs, antibodies	*E. coli*	2×10^5-10^5	10^4	-	+	-	-	8
Fluorescence detection	GO, QDs, antibodies	*E. coli*	10-10^7	5	-	+	-	-	9
Combination of RT-PCR and miniaturized NMR	Magneto-DNA	13 species of bacteria		single bacteria	-	+	-	-	10
Electrochemical immunosensor	Polytyramine, antibodies	*S. pyogenes*	10^4-10^7		-	-	-	-	11
Colorimetric	DNAzyme, urease	*E. coli*	5-5×10^7	1-100	+	+	-	-	12
Fluorescence imaging	Si nanowires, concanavalin A	*S. aureus*		10	-	+	-	+	13
Fluorescence detection	Diblock copolymers	*E. coli*		5.5×10^4	-	-	-	+	14
Fluorescence imaging, MR imaging	Amphiphilic copolymers	*E. coli*	0-4.5×10^7	8.5×10^5, 5×10^3	+	+	-	+	15
Sandwich ELISA	Artificial antibodies	*S. aureus*	10^4-10^7	5×10^2	+	+	+	+	Present assay

Reference

[1] a) A. Goux, M. Etienne, E. Aubert, C. Lecomte, J. Ghanbaja and A. Walcarius, *Chem. Mater.* 2009, **21**, 731-741; b) A. Walcarius, E. Sibottier, M. Etienne and J. Ghanbaja, *Nat. Mater.* 2007, **6**, 602-608.

[2] F. Wang and C. Mao, *Chem. Commun.* 2009, **45**, 1222-1224.

[3] G. Cheng, J.-L. Zhang, Y.-L. Liu, D.-H. Sun and J.-Z. Ni, *Chem. Commun.* 2011, **47**, 5732-5734.

[4] P. D. Josephy, T. Eling and R. P. Mason, *J. Biol. Chem.* 1982, **257**, 3669-3675.

G. Cheng, J.-L. Zhang, Y.-L. Liu, D.-H. Sun and J.-Z. Ni, *Chem. Commun.* **2011**, *47*, 5732-5734.

[5] O. R. Miranda, X. Li, L. Garcia-Gonzalez, Z.-J. Zhu, B. Yan, U. H. F. Bunz and V. M. Rotello, *J Am. Chem. Soc.*, 2011, **133**, 9650-9653.

[6] P. Laurino, R. Kikkeri, N. Azzouz and P. H. Seeberger, *Nano lett.*, 2011, **11**, 73-78.

[7] P.-H. Chan and Y.-C. Chen, *Anal. Chem.*, 2012, **84**, 8952-8956.

[8] X. Xu, Y. Chen, H. Wei, B. Xia, F. Liu and N. Li, *Anal. Chem.*, 2012, **84**, 9721-9728.

[9] E. Morales-Narváez, A.-R. Hassan and A. Merkoçi, *Angew. Chem. Int. Ed.*, 2013, **52**, 13779-13783.

[10] H. J. Chung, C. M. Castro, H. Im, H. Lee and R. Weissleder, *Nat. Nanotech.*, 2013, **8**, 369-375.

[11] A. Ahmed, J. V. Rushworth, J. D. Wright and P. A. Millner, *Anal. Chem.*, 2013, **85**, 12118-12125.

[12] K. Tram, P. Kanda, B. J. Salena, S. Huan and Y. Li, *Angew. Chem. Int. Ed.*, 2014, **53**, 12799-12802.

[13] Y. Q. Li, B. Zhu, Y. Li, W. R. Leow, R. Goh, B. Ma, E. Fong, M. Tang and X. Chen, *Angew. Chem. Int. Ed.*, 2014, **53**, 5837-5841.

[14] Y. Li, X. Hu, S. Tian, Y. Li, G. Zhang, G. Zhang and S. Liu, *Biomaterials*, 2014, **35**, 1618-1626.

[15] Y. Li, H. Yu, Y. Qian, J. Hu and S. Liu, *Adv. Mater.*, 2014, **26**, 6734-6741.
